# Time-dependent biphasic modulation of human BDNF by antidepressants in neuroblastoma cells

**DOI:** 10.1186/1471-2202-9-61

**Published:** 2008-07-05

**Authors:** Lorena Donnici, Ettore Tiraboschi, Daniela Tardito, Laura Musazzi, Giorgio Racagni, Maurizio Popoli

**Affiliations:** 1 Center of Neuropharmacology, Department of Pharmacological Sciences and Center of Excellence on Neurodegenerative Diseases, University of Milano, Italy; 2 Axxam SpA, San Raffaele Biomedical Science Park, Milano, Italy; 3 Sigrid Jusélius Laboratory, Neuroscience Center, University of Helsinki, Finland

## Abstract

**Background:**

Recent rodent studies reported that antidepressant treatments affect the expression of brain-derived neurotrophic factor (BDNF) mRNA in a way that is dependent on treatment duration, by selective modulation of different BDNF transcripts. However, no data are available for the human BDNF gene. We studied the effect of different antidepressants on BDNF mRNA expression in human neuroblastoma SH-SY5Y cells.

**Results:**

Cultured cells were treated with the antidepressants fluoxetine, reboxetine and desipramine for different time lengths (6, 24, 48 hours). Expression of total BDNF mRNA was analyzed by reverse transcription PCR and levels of different BDNF transcripts were detected by hemi-nested PCR with specific primers.

Short-term treatment (6 hours) with reboxetine or desipramine reduced total BDNF, whereas long-term treatment (48 hours) significantly increased total BDNF mRNA levels. These changes were accounted for by differential regulation of BDNF IV and VIa/b transcripts. Fluoxetine showed no significant effects.

**Conclusion:**

This is the first study showing biphasic changes in the expression of total and specific BDNF transcripts in human cells following antidepressant treatments. These findings suggest that biphasic induction of BDNF by antidepressants could be a feature common to rodents and humans and encourage the use of SH-SY5Y cells as a tool for investigation of drug effects on human genes.

## Background

Brain-derived neurotrophic factor (BDNF) has been implicated in both the pathophysiology and pharmacotherapy of depression [1-3]. It has been shown that BDNF expression and/or function is impaired in major depression or following stress paradigms, while it is up-regulated by physical exercise and antidepressants. However, different and sometimes conflicting findings have been reported [2], showing that antidepressants change total BDNF expression level depending on length of the treatment and time interval following administration. Indeed, independent investigations showed that short-term antidepressant treatment decreased BDNF expression in rodents, whereas long-term treatment increased BDNF [4-6].

The increasing knowledge of BDNF gene in rodents [7,8] has encouraged the research on the modulation of different BDNF transcripts by pharmacological treatments. Recent studies showed that different drugs, lengths of treatment and drug/physical exercise combination, as well as stress paradigms, may selectively influence the transcription of specific BDNF transcripts in rodents [6,9-12].

**Figure 1:**
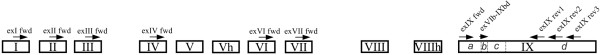
Schematic representation of human BDNF gene and of primers used.

However, to the best of our knowledge no data are available yet on the effect of antidepressants on human BDNF transcripts [13]. In addition, the gene structure of human BDNF has been recently revised. Accordingly, the human BDNF gene contains ten upstream untranslated exons (numbered I, II, III, IV, V, Vh, VI, VII, VIII, and VIIIh) that are alternatively spliced to a common downstream exon IX containing the coding region and the 3' untranslated region (see Figure 1). Therefore, aim of the present study was to investigate whether antidepressant treatments of different time lengths induce changes in the expression of BDNF gene also in cultured human cells and to assess whether these modifications could be explained by differential regulation of BDNF transcripts. Therefore, total BDNF mRNA and distinct BDNF transcript levels were measured by semi-quantitative PCR after treatment with different antidepressant drugs in human neuroblastoma SH-SY5Y cells.

### Experimental Procedures

#### Cell culture and pharmacological treatment

Human neuroblastoma SH-SY5Y cells were obtained from Interlab Cell Line Collection (Genova, Italy), at passage P14; only cells between passages P16 to P25 were used. Cells were grown in Minimum Essential Medium (Invitrogen, Carlsbad, CA), containing 10% foetal bovine serum, 2 mM glutamine and non-essential aminoacids (1 mg/l) in a humidified incubator (95% air, 5% CO_2_) at 37°C. Cells were treated for 6, 24 and 48 hours before harvesting (all cells were kept in culture for 3 days total) with different antidepressants: the selective serotonin reuptake inhibitor fluoxetine (FLX), the selective norepinephrine reuptake inhibitor reboxetine (RBX) and the tricyclic antidepressant desipramine (DMI), predominantly inhibiting norepinephrine reuptake (all 10 μM, final concentration).

#### RNA isolation, cDNA synthesis and reverse transcription-PCR for total BDNF expression

Cells from twelve independent experiments were lysed with Trizol (Invitrogen) and total RNA was isolated with Phase Lock Gel Heavy (Eppendorf, Hamburg, Germany). RNA purity was confirmed by spectrophotometry (A_260_/A_280_>1.7) and RNA integrity was visualized by agarose gel electrophoresis. 4 μg of RNA were reverse transcribed using cloned AMV first-strand synthesis kit (Invitrogen) and random hexamers. In order to detect total BDNF, PCR was performed with primers designed on the sequence of the coding exon, exon IX (exIX fwd and exIX rev3 primers) (Table 1 and Figure 1): 5 min 95°C, 33 cycles of 95°C for 15 s, 58°C for 10 s and 72°C for 30 s. BDNF exon IX primers were used in the same reaction tube together with primers for the housekeeping Ubiquitin C (UBC) gene [14] (Table 1), in order to co-amplify the target gene and the internal standard, which did not vary throughout experiments. The number of cycles and primer concentrations were chosen experimentally in order to fall into the exponential phases of the amplification reaction. For semi-quantitative analysis, amplicons were detected by gel electrophoresis (8% acrylamide, ethidium bromide staining). Bands were acquired with Bio-Rad GelDoc System (Bio-Rad Laboratories, CA) and intensities were measured with Quantity One software (Bio-Rad Laboratories). All the PCR amplicons were sequenced and found to mirror expected sequences for the different transcripts (not shown). The intensities of the PCR products of BDNF total mRNA were normalized on the intensity of the house-keeping gene UBC and then the data were reported as ratio between treated cells and control cells. For statistical analysis two-way analysis of variance (ANOVA) was performed followed by Bonferroni post-hoc tests.

**Table 1:** Sequences of primers used for RT-PCR.

**Primers**	**Sequence**
UBC fwd	atttgggtcgcggttcttg
exI fwd	cacttgagtctccaggacagc
exII fwd	caacggatttgtccgaggtgg
exIII fwd	atgcctcactgagcccagttcc
exIV fwd	cggagcagctgccttgatgg
exVIa/b fwd	ctggagccagaatcggaacc
exVII fwd	aacccacatctctacccatcc
exVIb-IXbd fwd	ggaagaaggagaacttgaagc
exIX fwd	actctggagagcgtgaatgg
UBC rev	tgccttgacattctcgatggt
exIX rev1	atccaacagctcttctatcacg
exIX rev2	atactgtcacacacgctcagc
exIX rev3	cgtagaagtattgcttcagttgg

#### RNA isolation, cDNA synthesis and reverse transcription-PCR for BDNF isoforms

RNA was extracted as above from cells of six independent experiments. 8 μg of RNA were reverse transcribed using cloned AMV first-strand synthesis kit (Invitrogen) and 2 pmoles of the BDNF specific primer exIX rev3 (Table 1), in order to increase the specificity of the reaction. Due to the low level of some transcripts, semi-quantitative hemi-nested PCR was used to detect specific BDNF exons [15]. Figure 1 and Tables 1 and 2 show human BDNF transcripts and primers used. To preamplify transcripts, a first PCR was performed (5 min 95°C, 15 cycles of 95°C for 15 s, 58°C for 10 s and 72°C for 30 s) using exIX rev2 and forward primers specific for individual BDNF transcripts. The second PCR was carried out with 2 μl of the first PCR products, using the reverse primer exIX rev1 and specific forward primers. The number of cycles and primer concentrations were chosen experimentally in order to fall into the exponential phases of the amplification reaction (between 20 and 30 cycles). UBC was used as internal control. Measurement of band intensities, normalization of data and statistical analysis were as described for total BDNF.

**Table 2:** Human BDNF transcripts and specific primers used for RT-PCR.

**Transcript**	**Accession number**	**Primers**	**Product (bp)**
BDNF I	EF689021.1	exI fwd/exIX rev1	269
BDNF II	EF674517.1; EF674518.1; EF674519.1	exII fwd/exIX rev1	301
BDNF III	EF674520.1	exIII fwd/exIX rev1	254
BDNF IV	EF674521.1	exIV fwd/exIX rev1	283
BDNF VIa/b	EF689014.1; EF689015.1	exVIa/b fwd/exIX rev1	306
BDNF VIb-IXbd	EF689016.1	exVIb-IXbd fwd/exIX rev1	296
BDNF VIIa/b	EF689017.1; EF689018.1	exVII fwd/exIX rev1	285

## Results

In order to characterize the effects of different antidepressants on total human BDNF mRNA expression, SH-SY5Y neuroblastoma cells were treated with FLX, RBX or DMI for 6 h (to assess the effects of short-term treatments), 24 or 48 hours, as in recent studies assessing the long-term effects of antidepressants in cultured cells [16,17]. With regard to the antidepressant concentration in the cell medium, several studies have found that therapeutic plasma levels of these drugs in humans under chronic treatment are in the low micromolar range. However, it has been also shown that their concentrations in brain tissue can be as much as 20 times higher compared to plasma levels [18,19]. Moreover, it has been reported that brain concentrations of different antidepressants administered systemically at different doses were similar, without a clear correlation with either treatment dose or treatment duration [19]. Therefore, in order to expose the neuroblastoma cells to a drug concentration as close as possible to those reached in brain tissue, we chose to treat cultured SH-SY5Y cells with 10 μM FLX, RBX or DMI in our experiments.

**Figure 2:**
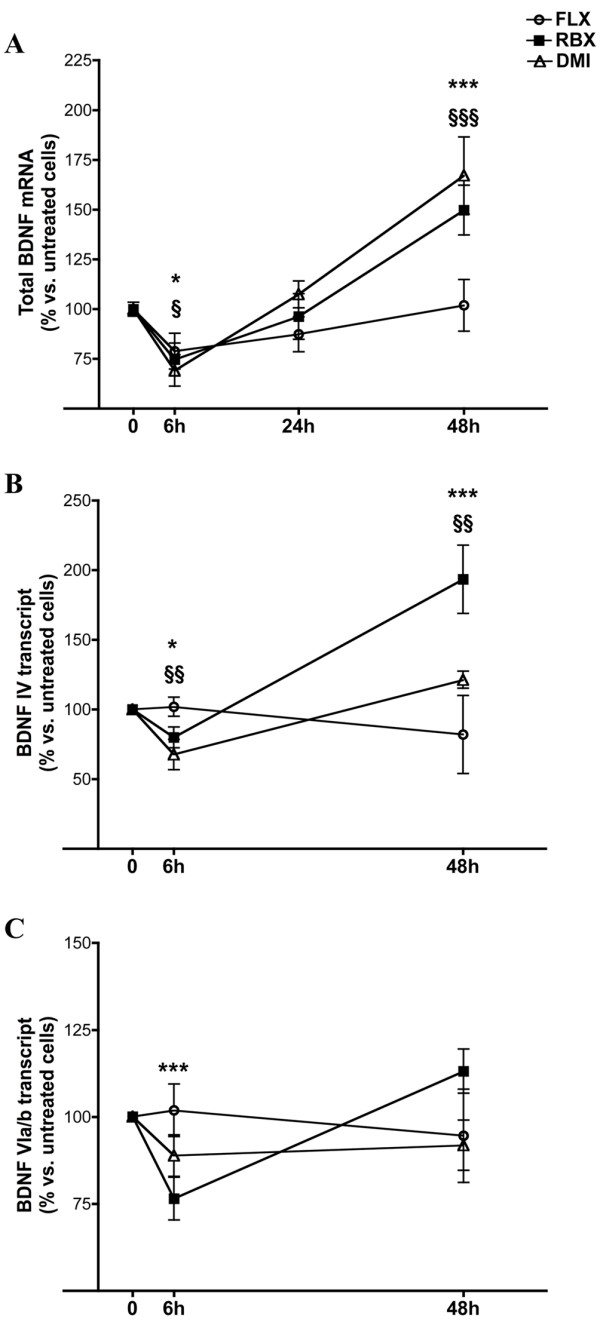
**Effects of antidepressant treatment on expression of total BDNF mRNA and BDNF transcripts in SH-SY5Y cells**. **A**. Expression of total BDNF mRNA in SH-SY5Y cells after 6 h, 24 h and 48 h treatment with 10 μM fluoxetine (FLX), reboxetine (RBX) or desipramine (DMI), relative to untreated cells. Data are expressed as % intensity units/mm^2 ^vs. untreated cells (mean ± s.e.m.). Statistics: two-way ANOVA, Bonferroni post-hoc test vs. untreated cells: *p < 0,05 ***p < 0,001 RBX vs. untreated cells; §p < 0,05 §§§p < 0,001 DMI vs. untreated cells. **B**. Expression level of BDNF IV transcript after 6 h and 48 h treatment with 10 μM FLX, RBX or DMI, relative to untreated cells. *p < 0.05 ***p < 0.001 RBX vs. untreated cells; §§p < 0.01 DMI vs. untreated cells. **C**. Expression level of BDNF VIa/b after treatment as in (B). Data and statistics as above. ***p < 0.001 RBX vs. untreated cells.

Two-way ANOVA analysis of total BDNF mRNA expression showed significant effects of time (F_3,193 _= 25.04; p < .0001), drug (F_2,193 _= 4.60; p < .05) and interaction between the two variables (F_6,193 _= 4.48; p < .001). In particular, RBX and DMI exerted a biphasic effect on BDNF mRNA: BDNF mRNA expression was reduced after 6 hours treatment with RBX or DMI, returned to basal level at 24 hours and was significantly increased after 48 hours (Fig. 2A). FLX did not show any significant effect at any time.

The BDNF transcript expression pattern in SH-SY5Y cells is similar to that of human cortical tissue [20]: according to a recent nomenclature of BDNF gene [13], BDNF IV and VI are the most highly expressed transcripts, representing over 80% of the total BDNF. Therefore, in order to evaluate if the modifications induced in total BDNF mRNA levels by antidepressants were due to alterations in the main BDNF transcripts, the expression profile of BDNF IV and VIa/b transcripts were analyzed. Cells were treated with FLX, DMI or RBX for 6 hours or 48 hours, times at which expression of total BDNF mRNA was changed after antidepressants (Fig. 2A), and BDNF transcript expression level was detected with semi-quantitative hemi-nested PCR. Two way ANOVA showed significant effects of time (F_2,144 _= 37,42; p < .0001), drug (F_2,144 _= 23,39; p < .0001) and time/drug interaction (F_4,144 _= 26,94; p < .0001) for BDNF IV transcript: BDNF IV was significantly reduced after 6 hours and significantly increased after 48 h of RBX or DMI (Fig. 2B). FLX showed no effect. Two way ANOVA of BDNF VIa/b (Fig. 2C) showed a significant effect of time (F_2,107 _= 7,19; p < .05) and of time/drug interaction (F_4,107 _= 7,53; p < .0001). Post-hoc comparisons showed a significant reduction of BDNF VIa/b after 6 hours of RBX and no effects after 48 hours (Fig. 2C). In addition, we analyzed the expression of the low-level BDNF transcripts, BDNF I, II, VIb-IXbd and VIIa/b; all drug treatments did not change the expression of the transcripts (data not shown). BDNF III was undetectable; expression of exons V, Vh, VIII and VIIIh was not measured because, when the present work was completed, the new nomenclature was not yet published [13].

## Discussion

To the best of our knowledge, this is the first study analyzing the effect of antidepressant treatments on the expression of BDNF transcripts in cells of human origin. Previous studies found time-dependent biphasic changes in the expression of total BDNF induced by antidepressants in rat brain [4-6]. Similarly, we found that in SH-SY5Y cells RBX and DMI induced biphasic changes in total BDNF mRNA expression. Indeed, short-term treatment with both antidepressants significantly reduced, whereas long-term treatment increased total BDNF mRNA. At the intermediate time BDNF mRNA levels were similar to control. FLX did not significantly affect BDNF expression, likely owing to the absence of the serotonin transporter in SH-SY5Y cells, which instead express the norepinephrine transporter [21,22], and the 5-HT_2b _serotonergic receptor and the α_2c _adrenoreceptor [23-26]. Indeed, we could confirm the absence of any signal for serotonin transporter mRNA in SH-SY5Y cells by PCR (not shown). An alternative explanation could be found in the lack of changes in BDNF expression after fluoxetine treatment in rodents, as reported by several studies [9,27-30].

The present results suggest that early reduction of total BDNF mRNA in SH-SY5Y cells was mainly accounted for by a reduction of BDNF IV by RBX and DMI administration, and of BDNF VIa/b by RBX. On the other hand, the increase in total BDNF mRNA after 48 hours of treatment appeared to be mainly accounted for by the induction of BDNF IV by both drugs.

Interestingly, previous studies in rodents have shown a differential regulation of distinct BDNF transcripts by antidepressant treatments [6,9,11]. In particular, it has been shown that chronic defeat stress, a model of depression, selectively down-regulated in mouse hippocampus the expression of BDNF III and BDNF IV transcripts (corresponding in the present study to BDNF IV and BDNF VIa/b respectively, according to the new nomenclature of BDNF gene), while chronic imipramine treatment reversed this down-regulation [12].

The underlying molecular mechanisms are at present unknown; however, recent evidence from several groups suggest that different signaling mechanisms may be responsible for the regulation of BDNF transcription [2]. As an example, it has been shown that different BDNF promoters contain multiple sites for various transcription factors (i.e. CREB, CaRF), responding to different signaling pathways mainly activated by calcium fluxes (CaM kinase cascades), by cyclic AMP (cAMP-PKA cascade) and by neurotrophic factors (MAP-Erk pathway). In this regard, we have recently reported that chronic administration of different antidepressants in rats exerts distinct actions on CREB activation that seem to depend on a differential modulation of CaM kinase IV and MAPK-Erk1/2 cascades [31]. Furthermore, we found that the CaM kinase IV cascade is also involved in the regulation of CREB activation by the mood stabilizer lithium [32].

Overall, although further studies are needed to clarify the upstream mechanisms, these results suggest that time-dependent biphasic changes in the expression of BDNF IV and VIa/b account for the observed changes induced by antidepressants in total BDNF mRNA of SH-SY5Y cells. Validation by quantitative Real-Time PCR is warranted. A limitation of the present work is that BDNF protein was not measured; because often mRNA and protein expression do not correlate, measurement of BDNF protein in SH-SY5Y after antidepressant treatments is warranted. Also considering recent reports on the different structure and regulation between rodent and human BDNF gene [7,13], SH-SY5Y cells could provide a useful tool to screen the effects of different psychotropics on human BDNF. Furthermore, it will be interesting to investigate whether similar effects are also found in peripheral cells of patients treated with antidepressants. In this regard, it is noteworthy that changes in serum concentrations of BDNF after antidepressant treatments have been measured in a number of works (as an example, see ref [33]).

## Conclusion

The present findings show biphasic changes in the expression of total and specific BDNF transcripts following antidepressant treatments in human cells and encourage the use of SH-SY5Y cells as a tool for research of psychotropic drug effects on human genes.

## Authors' contributions

LD carried out the molecular biology work. ET carried out the molecular biology work. DT performed the statistical analysis, and drafted the manuscript. LM performed the statistical analysis, and drafted the manuscript. GR help to draft the manuscript and provided useful discussion. MP designed the study, and drafted the manuscript. All authors contributed to and have approved the final manuscript.
